# P-817. Can We Stop Taking Follow-Up Blood Cultures for *Brucella Melitensis* in Hospitalized Adults?

**DOI:** 10.1093/ofid/ofae631.1009

**Published:** 2025-01-29

**Authors:** Daniel Grupel, Meir Cherniak, Matan Cohen

**Affiliations:** Hadassah Medical Center and the Hebrew University, Jerusalem, Jerusalem, Yerushalayim, Israel; Hadassah Hebrew University Medical Center, Jerusalem, Yerushalayim, Israel; Clalit Health Services, Jerusalem district, Bet Shemesh, Yerushalayim, Israel

## Abstract

**Background:**

*Brucella melitensis* is an important zoonotic pathogen causing human disease worldwide. Blood cultures are often essential in establishing the diagnosis in hospitalized patients.

The need for repeated blood cultures is well established in gram-positive bacteremia and is still debated in gram-negative bacteremia. There is scant data regarding the need for repeated blood cultures in brucellosis. Follow-up blood cultures (FUBCs) may direct physicians to cryptogenic focal infection but are also associated with multiple venipunctures, prolonged hospitalization, and contamination. Moreover, repeatedly positive blood cultures can cause clinicians to pursue excessive workups.

**Figure:**
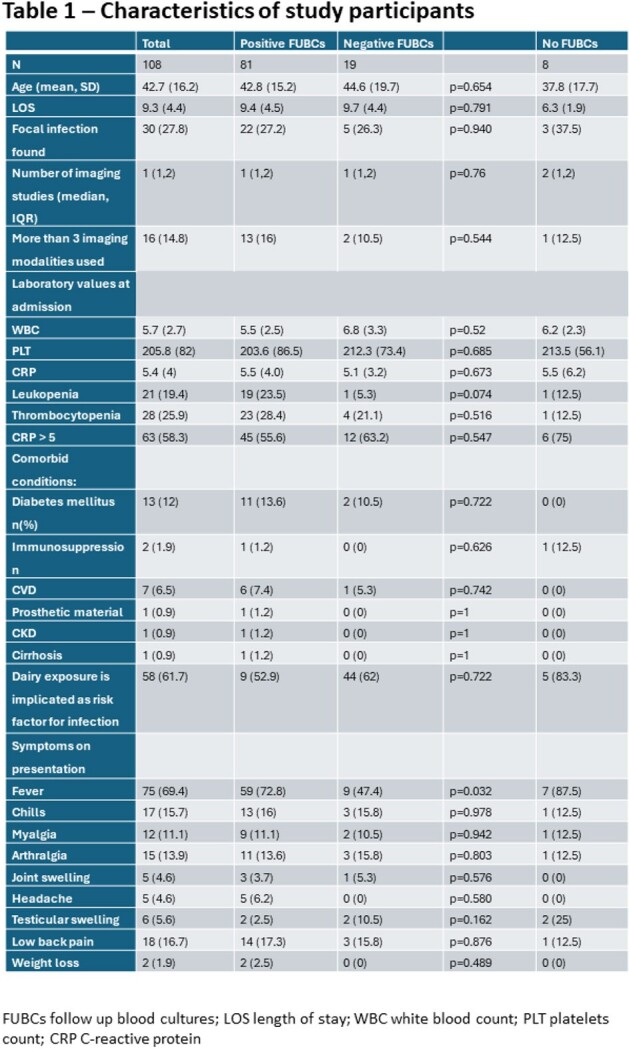

**Methods:**

We retrospectively collected data on all positive cultures growing *Brucella sp*. collected between 1/10 and 12/22 in adults at the Hadassah Medical Center campuses.

The primary outcome was brucellosis relapse or treatment failure. Hospitalization resource utilization, treatment modifications and length of stay were also recorded.

Three groups were defined- patients with 1) a single blood culture positive for Brucella without FUBC; 2) a first positive blood culture with negative FUBCs; and 3) positive FUBCs. Subgroup analysis was performed according to the positivity of the last culture taken during hospitalization.

**Results:**

We found 525 cultures taken from 176 patients positive for *Brucella sp*. 68 patients met the exclusion criteria. 7.5% of the cohort had a single culture, 17.8% had negative FUBCs, and 74.8% had positive FUBCs.

No significant difference was observed in the primary outcome between the groups with positive or negative FUBCs (15.8% and 11.3%, respectively, p=0.695). The clinical presentation, length of stay, number of imaging modalities used, and rates of treatment modification also did not differ between the groups (Table 1).

**Conclusion:**

In our brucellosis cohort, FUBC positivity was not associated with an increased rate of complications or adverse events despite similar baseline characteristics and clinical course.

Furthermore, the groups with FUBCs did not fare differently than those without FUBCs.

With the caveats of an observational study, these data suggest that discontinuing FUBCs in hospitalized brucellosis patients should be considered as it seems reasonable and safe.

**Disclosures:**

**All Authors**: No reported disclosures

